# Printed Electrodes in Microfluidic Arrays for Cancer Biomarker Protein Detection

**DOI:** 10.3390/bios10090115

**Published:** 2020-09-07

**Authors:** Lasangi Dhanapala, Colleen E. Krause, Abby L. Jones, James F. Rusling

**Affiliations:** 1 Department of Chemistry, University of Connecticut, 55 North Eagleville Road, Storrs, CT 06269, USA; lasangi.dhanapala@uconn.edu (L.D.); abby.jones@uconn.edu (A.L.J.); 2 Department of Chemistry, University of Hartford, Bloomfield Ave., West Hartford, CT 06117, USA; ckrause@hartford.edu; 3 Department of Surgery and Neag Cancer Center, UConn Health, Farmington, CT 06232, USA; 4 Institute of Materials Science, University of Connecticut, 97 N. Eagleville Road, Storrs, CT 06269, USA; 5 School of Chemistry, National University of Ireland Galway, University Road, H91 TK33 Galway, Ireland

**Keywords:** screen-printed electrodes, inkjet-printed electrodes, immunoassays, cancer, biomarkers, protein

## Abstract

Medical diagnostics is trending towards a more personalized future approach in which multiple tests can be digitized into patient records. In cancer diagnostics, patients can be tested for individual protein and genomic biomarkers that detect cancers at very early stages and also be used to monitor cancer progression or remission during therapy. These data can then be incorporated into patient records that could be easily accessed on a cell phone by a health care professional or the patients themselves on demand. Data on protein biomarkers have a large potential to be measured in point-of-care devices, particularly diagnostic panels that could provide a continually updated, personalized record of a disease like cancer. Electrochemical immunoassays have been popular among protein detection methods due to their inherent high sensitivity and ease of coupling with screen-printed and inkjet-printed electrodes. Integrated chips featuring these kinds of electrodes can be built at low cost and designed for ease of automation. Enzyme-linked immunosorbent assay (ELISA) features are adopted in most of these ultrasensitive detection systems, with microfluidics allowing easy manipulation and good fluid dynamics to deliver reagents and detect the desired proteins. Several of these ultrasensitive systems have detected biomarker panels ranging from four to eight proteins, which in many cases when a specific cancer is suspected may be sufficient. However, a grand challenge lies in engineering microfluidic-printed electrode devices for the simultaneous detection of larger protein panels (e.g., 50–100) that could be used to test for many types of cancers, as well as other diseases for truly personalized care.

## 1. Introduction

Growing rates of incidence and mortality have made cancer a global pandemic, a key impediment for increasing life expectancy and quality of life [1,2]. Most of the current cancer diagnostic tests rely on tissue biopsies and imaging techniques. First of all, these techniques require a physical tumor to be detectable. Biopsies analyze tissue morphology and cellular arrangement, but the precise location of the tumor is needed, they are highly invasive and can easily miss tumor cells. Imaging techniques such as mammograms or colonoscopies only allow tumor detection and do not allow early diagnosis before the onset of tumor development [3,4]. These techniques are relatively expensive and require expert technical knowledge for the test and its interpretation (especially for mammograms), limiting their widespread accessibility. Relying only on these tests, cancer cells can spread through the body before being diagnosed, drastically reducing survival rates [1,5,6]. Thus, a new strategy has been developing for personalized cancer diagnosis which will enable patients to access health or disease conditions early. This approach will rely on fast, quick, sensitive, and accurate assays with no or minimum invasion, utilizing samples such as blood, urine, and saliva. Full implementation of this approach, which has thus far been very slow to take hold in the clinical realm, has the potential to greatly improve rates of survival and quality of life of millions of cancer patients across the globe. 

**Figure 1:**
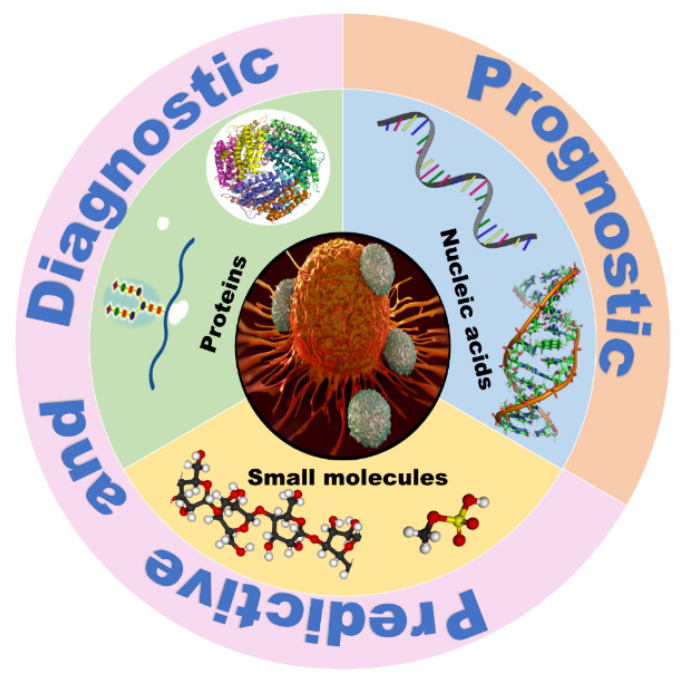
A simple overview of cancer biomarkers and their clinical importance.

Cancer progression is marked by abnormal cell division, followed by the expression of specific molecules (biomarkers) that are otherwise absent or typically expressed in small amounts in healthy cells [7,8,9,10]. A biomarker is an indicator of a person’s health, disease condition, or responses to therapy and collective of a broad range of biomolecules from proteins, glycoproteins, nucleic acids to a variety of other small molecules found in bodily fluids [3,4,6,8,9,10,11]. Nucleic acids such as DNA and RNA mainly act as prognostic biomarkers providing a risk assessment of cancer [4,9], whereas protein biomarkers serve as diagnostic/predictive biomarkers of cancer, to provide a quick picture of the patient’s health [9,12,13]. The most accurate way to use these tools is to measure multiple biomarkers for each type of risk, or in the case type of cancer (Figure 1). Thus, the majority of cancer biomarker research focuses on protein detection for early diagnosis, post-surgery reoccurrence, and cancer staging [5,9].

Despite significant research gains in this area, only a handful of protein cancer biomarkers and no protein panels are approved by the US Food and Drug Administration (FDA) [3,5,14,15] (Table 1) for clinical practice. This discrepancy between research and clinical practice is due to three main factors: (a) Lag in development of point-of-care (POC) devices for detection of panels of biomarkers [5,9,13] (b) lack of sufficient National Institutes of Health (NIH) funding for translational research in development and validation of effective biomarker panels, and (c) inability to detect rare biomarkers due to poor sensitivity for low concentration biomarkers coupled in a panel with more abundant proteins [16,17,18,19]. Detection of a panel of biomarkers enables better diagnostic accuracy than single proteins. Current commercial assays, including multiplexed enzyme-linked immunosorbent assay (ELISA), LC-MS/MS [5,9,19], mesoscale electrochemiluminescence (ECL) [20], luminex [19,21], and single-molecule counting system Simoa-HD [22], struggle to truly answer the above challenges, slowing translation of protein biomarker research into clinical practice.

The inherent sensitivity and ease of multiplexing of electrochemical immunosensors has placed them among the most utilized methods of biosensors. Coupled with simplicity of instrumentation, low cost, the capability of miniaturization, automation, and ease of integration to microfluidic devices has made electrochemical immunosensors an excellent platform for fabrication of POC devices [24,25]. The detection ability of each electrode-sensor is based on the desired function of a specific electrode, the electrode material, surface modification, and dimensions [26]. Over the past several decades miniaturization and the production cost of electrodes that make up the electrochemical sensor platform has improved with the advent of a variety of thin-film technologies. Common methods in developing these thin-film electronic devices include chemical vapor deposition (CVD) [27], photolithography [28], stencil printing [29], screen printing [30], and inkjet printing [31,32]. Inkjet printing has also been used to make flexible thin-film transistors that can be used as sensors [33]. All these methods allow for well-defined electrode fabrication and insulation. However, in terms of mass production, screen-printing and inkjet printing have emerged to become the favored approach to produce disposable electrochemical sensing platforms and arrays [9,34,35,36]. 

In this review, we cover some of the key cancer biomarker research done using printed electrodes focusing on the evolution of immunosensors invented by our group, as well as examples from recent literature landmarks of printed electrode biosensors. We focus mainly on screen-printed electrodes (SPEs) with a few illustrations of inkjet-printed electrode sensors. The primary focus on SPEs is driven by the numerous applications of SPEs to POC devices, including the poster-child of biosensors—the glucose biosensor [25].

## 2. Immunoassay Techniques for Printed Electrodes

### 2.1. Immunoassay Protocol

**Scheme 1:**
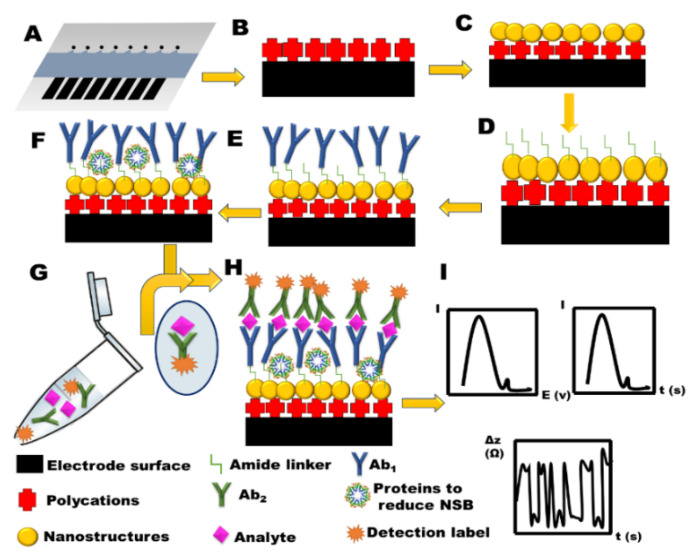
Representation of the general protocol followed (**A**–**I**) during an electrochemical immunoassay. (**A**) A typical screen-printed carbon electrode, (**B**) Surface modification by LBL where polycations are coated on the electrode surface, (**C**) Equipping the surface with nanostructures, (**D**) Amidization reaction with surface functional group via linkers such as EDC 1-(3-(Dimethylamino)propyl)-3-ethyl carbodiimide hydrochloride (EDC) and N-hydroxysulfosuccinimide (NHSS) [17], (**E**) Ab_1_ immobilization followed by incubation, (**F**) Reduction of NSB by washing with detergents and BSA or casein proteins, (**G**) Introduction of a signal generating labeled Ab_2_ for detection of analyte, (**H**) Final arrangement of Ab_1,_ Ag and Ab_2_ to give a sandwich type ELISA, (**I**) Electrochemical detection through voltammetry, amperometry, or impedance.

Heineman and coworkers pioneered the introduction of ELISA-type techniques into electrochemical biosensors, allowing a floodgate of research that led to modern electrochemical immunoassays [37]. Many immunoassays on printed electrodes adopted the sandwich ELISA platform, where the capture or primary antibodies (Ab_1_) are bound onto the sensor electrode surface and capture the protein antigens (Ag), which then entrap a detection or secondary antibody (Ab_2_), providing, in many cases, higher sensitivity and selectivity than classical optical detection ELISA and related techniques [3] (Scheme 1H). When coupled with microfluidics, electrochemical immunosensors can surpass the traditional drawbacks associated with ELISA to build a diagnosis platform at a lower sample volume, low reagent consumption, more efficient mixing, faster response times, and continuous monitoring [38,39,40,41].

**Figure 2:**
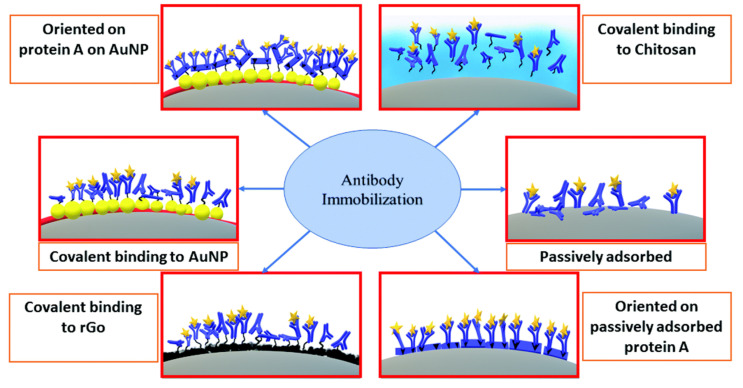
Different Ab_1_ immobilization strategies on SPCE. Reprinted with permission from [47], copyright 2019 The Royal Society of Chemistry.

Layer-by-layer (LBL) techniques have gained popularity in biosensor fabrication, following its introduction by Lvov and Decher in the early 1990s, and have been used frequently for the attachment of Ab_1_ on printed electrodes [42,43,44,45] (Scheme 1B). Carbon electrode surfaces carry a net negative charge in neutral solutions, and metal electrodes can be derivatized with anionic thiols, like mercapto-propionic acid, to give them a negative charge at pH < 6 [43]. Hence these negative surfaces can be layered with adsorbed polycations such as polyethyleneimine (PEI), poly(diallyldimethylammonium chloride) (PDDA), or poly(allylamine hydrochloride) (PAH) [44] (Scheme 1B). Then, the negatively charged nanomaterials can be adsorbed as the next layer (see Section 3.1 for use of nanomaterials in surface modification of electrodes) (Scheme 1C), followed with Ab_1_ immobilization, commonly through amidization [46] (Scheme 1D,E). Sharafeldin et al. further discussed different strategies of Ab_1_ immobilization on screen-printed carbon electrodes (SPCEs), such as (a) electrochemical adsorption of glutathione-coated gold nanoparticles (GSH-AuNP); (b) electrochemical deposition of graphene; (c) passive adsorption of antibodies directly to the carbon surface through the Fc region, with Fab region orientated towards the antigen-binding; (d) passive adsorption with random orientation; (e) chitosan films; and (f) covalent bonding of antibodies to GSH-AuNP. According to their study, sensitivity of the sensor mainly depends on the active area and antibody coverage enhanced by nanostructures (discussed later in detail), whereas a greater degree of orientation will extend the linear dynamic range to higher concentrations but not lower detection limits [47] (Figure 2).

**Figure 3:**
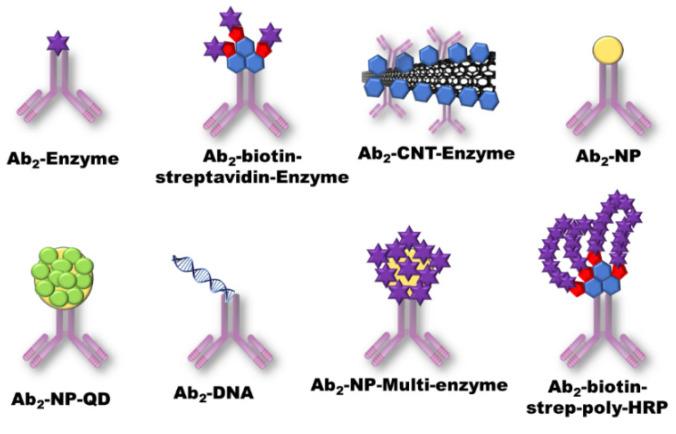
Different labels conjugated with the secondary antibody (Ab_2_) during electro immunoassays performed on printed electrodes.

Following Ab_1_ immobilization, analyte protein is introduced into a solution contacting the sensor, which then selectively captures Ab_2_, which is often accompanied by a detection label [9,10,11] (Figure 3). Labels can be anything that can produce a signal, including conductive polymers, nanoparticles, electroactive metal ions and complexes, and liposomes with electroactive species. Very often enzyme labels are used for detection [6,9,10,11,48]. Commonly, enzymes such as horseradish peroxidase (HRP), glucose oxidase, or alkaline phosphate (ALP) are used as labels to provide a high, reproducible, stable signal amplification [45]. A considerable amount of recent research focuses on the use of Ab_2_-HRP conjugates to immobilize the labels on the electrodes to avoid diffusional cross-talk between sensor elements [6]. Multiple HPR labels associated with one Ab_2_ can also enhance sensitivity tremendously, and, in some cases, nanoparticles have been used to bring Ab_2_ and HRP or other labels together [9,10]. Ab_2_-HRP can be achieved by streptavidin–biotin association with most of the Ab_2_ now commercially available in biotinylated form, or can easily be biotinylated using commercial kits [49,50,51]. Other similar associations with biotin include avidin and neutravidin [52,53]. Avidin tends to increase non-specific binding (NSB) due to its basic isoelectric pH (10.5), making it highly positively charged in physiological pH, hence, interacting with negatively charged surfaces [54]. Neutravidin is a promising alternative over both streptavidin and avidin due to its higher association with biotin and lower NSB [49,55]. 

Typically, after the immobilization of Ab_1_ and the analytes captured, the electrodes are washed by detergent and/or an NSB blocking protein, such as bovine serum albumin (BSA) or casein, to reduce the NSB that greatly decreases the signal/noise (S/N) ratio [9,10,11,17,24]. (Scheme 1F) NSB arises when the Ab_2_ molecules bind to non-antigen sites of the electrodes, providing a signal which is not proportionate to the analyte concentration, increasing the LOD and damaging the sensitivity. Casein and BSA are used to prevent NSB by sterically blocking the non-analyte sites, whereas detergent such as phosphate-buffered saline-Tween 20 (PBS-T20) or Tris buffer solution (TBS), with sodium dodecyl sulfate (SDS) or Triton 100-X, help to wash away weakly-bound Ab_2_ on non-analyte sites [17,56]. Another approach on reduction of NSB is through chemical attachments of the electrode surface to reduce protein adsorption through (a) polymerization strategies (polyethylene glycol (PEG), conducting polymers), (b) modification of allotropic carbons (carbon nanotubes), (c) sol-gel modification, (d) surface modification by diazonium salts, (e) metal nanoparticles (magnetic beads, gold and silver nanoparticles), (f) self-assembled monolayers (SAMs) [52]. In our hands, these chemical modifications are not so efficient in reducing NSB, and detergent and BSA or casein are still needed [6,9,10].

The signal generated by Ab_1_-Ag-Ab_2_ conjugation on the sensor may be detected through a variety of electrochemical techniques, including amperometry, voltammetry, square wave voltammetry (SWV), differential pulse voltammetry (DPV), stripping voltammetry, cyclic voltammetry, and impedance [11] (Scheme 1I).

### 2.2. Ultrasensitive Detection

The signal amplification provided through the enzyme label alone is not sufficient to reach the ultra-sensitivities required for many clinical cancer biomarker diagnoses. Thus, additional amplification strategies have to be adopted on the printed electrode systems [3,6,9,10,11]. Approaches include (a) redox cycling by using reversible redox couples regenerated electrochemically, chemically, or enzymatically (discussed in detail by Yang et al.) [48]; (b) multiple labeling such as multi-enzyme nanoparticles or polymers that provide a manifold of electrochemical events per bound analyte [48]. Other approaches include secondary antibody tags with dissolvable nanoparticles and multi-enzyme Ab_2_ conjugates (Figure 3) [9,10,11,17,48]. 

#### 2.2.1. Dissolvable Nanoparticles 

Dissolvable nanoparticle (NP) labeling has been used in biosensor development due to ease of mass-scale synthesis, chemical stability, high surface: volume ratio, low cost, and higher stability over enzyme labels, increasing the shelf life of the sensor [57,58,59]. Replacement of enzymes by NPs has opened a new chapter of research coined “nanozymes”. Examples include metal NPs, quantum dots (QDs), and most recently, carbon-based nanoparticles [57,60,61,62].

**Figure 4:**
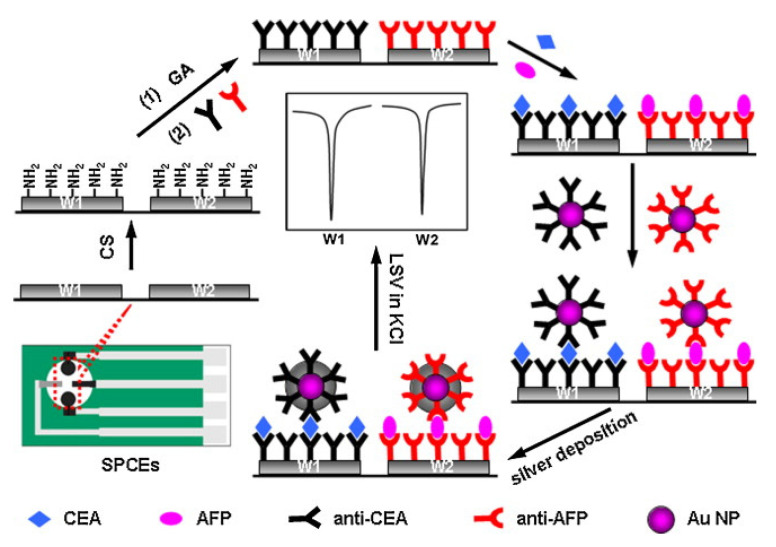
Schematic diagram of the assay protocol for multiplexed detection of AFP and CEA tumor biomarkers through silver NP reduction through AuNP-Ab_2_ conjugates. Reprinted with permission from [68], copyright 2012 Elsevier B.V.

Pioneering work on metal NPs on SPEs was done by Dequaire et al. using colloidal gold particles tagged with Ab_2_, following acid digestion and detection of the produced Au (III) particles via anodic stripping voltammetry (ASV) detecting immunoglobulin G (IgG) at μg mL^−1^ LODs [63]. Following, Joe Wang and his team did ground-breaking research on metal NPs for protein detection during the 2000s, including cyclic accumulation of gold nanoparticles to catalyze the precipitation of silver [64,65,66,67]. AuNPs were used by Lai at el. with a less destructive mode for multiplexed detection of α-fetoprotein (AFP) and carcinoembryonic antigen (CEA) with 3.9 and 3.5 pg mL^−1^ LODs, respectively. After immobilization of Ab_1_ on the electrode surface using chitosan linking, electrodes were incubated with AFP and CEA, followed by secondary antibodies anti-AFP and anti-CEA conjugated with AuNP tags. Next, silver nanoparticles (AgNPs) solution was deposited on the SPE and the Ag reduction was detected by linear sweep voltammetry (LVS) from −0.15 to 0.25 V at 50 mV s^−1^ in 1.0 M KCl solution [68] (Figure 4). Following this, a variety of NPs were used for protein biomarker detection, including silver NPS [68,69,70], platinum NPs [71,72,73,74], palladium NPs [57,75], copper NPs [76], and iridium NPs [57,77]. 

Additionally, multi-metal NP tags and hybrids of different metals are being reported [57,75]. Kalyoncu and coworkers used three different hybrid nanotags CuAu@ɣFe_2_O_3,_ ZnAu@ɣFe_2_O_3,_ and PbAu@ɣFe_2_O_3_ for multiplexed detection of vascular endothelial growth factor (VEGF), AFP, and CEA by binding Ab_2_ molecules with the respective nanotags. DPV was used for the detection of the oxidation peaks of Cu, Zn, and Pb ions obtained by acid dissolution of the hybrid NPS at +0.8, +1.3, and +0.1 V, respectively [78] A similar study was done by Putnin et al. with polyethylenimine-coated gold nanoparticles (PEI-AuNPs) conjugated with four different electroactive metal ions—Cd (II), Ag (I), Pb (II), and Cu (II)—as labels. They also used the same PEI-AuNPs for surface modification of the SPEs and detected four tumor biomarkers—PSA, AFP, CEA, and interleukin-8 (IL-8)—with LODs from 0.9 to 1.7 fg mL^−1^ [79].

QDs are attractive for designing bar-coded labels for multiplexed detection of biomarkers by differential tuning of the potentials specific for each QD [57,75,80,81]. In principle, such barcodes could be used for the simultaneous detection of thousands of biomarkers using computational deconvolution. The most widespread practice in detecting QDs is through ASV proceeded by acid digestion [82,83]. Merkoci et al. were among the first to demonstrate a less aggressive strategy by direct detection of CdS through the redox cycling of Cd (II). Water-soluble, glutathione-modified CdS QDs were introduced onto the electrode surface, followed by a deposition potential of −1.1 V for 120 s to reduce the Cd(II) in QDs to Cd(0), and SWV detection with a potential ranging from −1.1 to −0.7 V to oxidize the accumulated Cd(0) [84].

**Figure 5:**
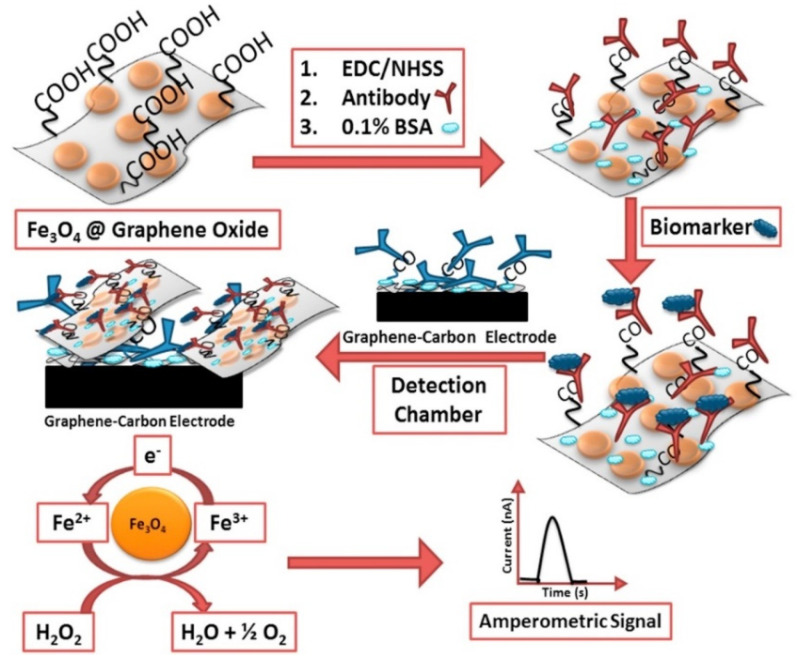
Schematic illustration of protein capture and detection by Fe_3_O_4_@GO sheets. After the analyte is captured by the GO sheets, it is captured on the SPCE surface coated with Ab_2_ and an amperometric signal is generated using H_2_O_2_. Reprinted with permission from [90], copyright 2016 Elsevier B.V.

Carbon-based nanoparticles became a “hot topic” after the discovery of graphene that worked its way into immunosensors. Graphene, fullerenes, and carbon nanotubes (CNTs) are among the most used carbon-based NPs [75]. Wang and coworkers pioneered the use of CNTs in immunosensors for the detection of DNA and proteins [85] and upright high surface area carbon nanotube forest sensors, with Ab_1_ attached at their ends, were developed by our research team [86,87,88,89] for detection of tumor biomarkers on pyrolytic graphite electrodes, and can be easily adapted to printed electrodes. More recently, we used graphene oxide (GO) nanosheets on SPEs to detect prostate-specific antigen (PSA) and prostate-specific membrane antigen (PSMA) with LODs of 5–15 fg mL^−1^. Mediator-free, Ab_2−_Fe_3_O_4_@GO particles were used as labels, and Fe_3_O_4_ shows peroxidase activity mimicking HRP, and reduces H_2_O_2_ to generate amperometric current at −0.3 V vs. Ag/AgCl in a microfluidic device. GO nanosheets are bound with multiple Fe_3_O_4_ nanoparticles to obtain paramagnetic Fe_3_O_4_@GO, to which Ab_2_ are immobilized using EDC/NHSS amidation to obtain Ab_2−_Fe_3_O_4_@GO, which first captures the biomarkers. This system is then introduced to a microfluidic device housing screen-printed carbon (Kanichi) arrays decorated with electrochemically-reduced graphene oxide (ERGO) immobilized with Ab_1_. The dynamic range was tunable based on the concentration of Fe_3_O_4_@GO used [90] (Figure 5). Pingarrón’s group designed a nanotag with graphene quantum dots (GQDs) and multiwalled carbon nanotubes (MWCNTs) hybridization for the detection of two metastatic proteins, IL-13 receptor-α2 (IL-13Rα2) and cadherin-17 (CDH-17), with sub-ng mL^−1^ detection using SPCE modified with p-aminobenzoic acid for covalent immobilization of Ab_1_. Assays were performed for 3 h with 0.5 μg of sample [91].

#### 2.2.2. Multi-Enzyme Conjugates

HRP and ALP are frequently used for multi-enzyme labels in conjugates, such as Ab_2_-magnetic beads, carbon-based multi-enzymes (Carbon nanotubes, graphene, carbon spheres), silica nanoparticles, metal nanoparticles (Au, Ag), and polymerized enzymes. Generally, these multi-enzyme tags require a mediator for their effective functionality as the bulky particles provide a steric and distance barrier for direct electron transfer between the enzyme and the sensor electrode surface. Hydroquinone is a frequently used mediator coupled with H_2_O_2_ that is being used on SPE systems [17,48,82,84].

**Figure 6:**
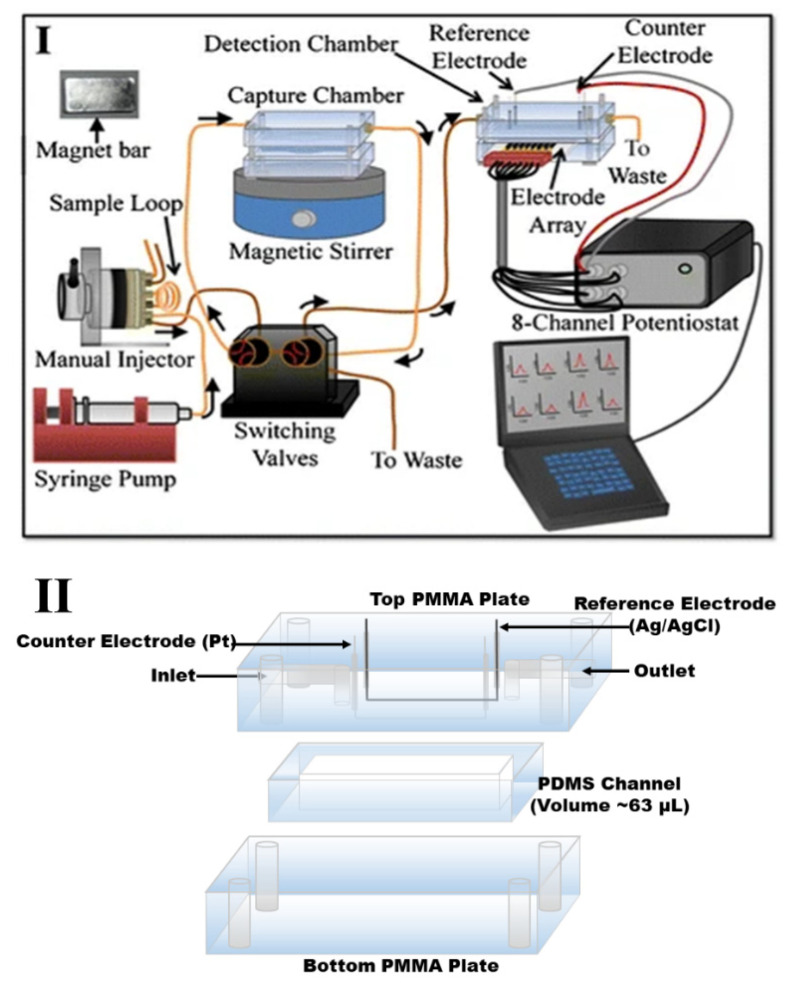
The microfluidic set-up used by our group in electroimmunosensors. (**I**) Schematic diagram of the pump (used for the flow of wash buffers and hydroquinone mediator into the detection chamber), HPLC injector (used for the injection of the sample into the detection chamber), switching valve (for alternation of the direction of fluids into the detection chamber, capture chamber or the waste), microfluidic chamber (houses the electrodes and the detection chamber), and the eight-channel multipotentionstat for amperometric detection of the analytes. (**II**) The microfluidic chamber with a poly (dimethoxy) silane channel (1.5 mm × 2.8 cm) sandwiched between two hard poly (methylmetacrylate) (PMMA) plates. The upper PMMA plate houses the platinum counter electrode and the Ag/AgCl reference electrode. Reprinted with permission from [101], copyright 2015 Springer Nature.

Magnetic beads (MBs) have drawn attention to the conjugation of multiple enzymes due to advantages over other nanoparticles such as ease of separation and ease of bioconjugation through the surrounding polymer layer [92]. Our research team exploited MB-based nanotags for the detection of various cancer biomarkers proteins. In our first paper using MBs in 2009 with Mani et al., we developed a sensitive assay for PSA with 7500 HRP molecules per MB to attain LODs of 0.5 pg mL^−1^ [93]. Later, collaborations with Munge et al. synthesized MBs with about 50,000 HRP enzymes, pushing LODs down to 1 fg mL^−1^ for interleukin 8 (IL-8) detection [94]. These studies were then extended for ultrasensitive single and multiplexed detection of cancer biomarker proteins on both SPEs and inkjet-printed electrodes coupled in microfluidic devices [31,95,96,97,98,99,100,101,102,103] (Figure 6).

Clinical diagnosis of oral cancer through the multiplexed detection of IL-6, IL-8, VEGF-C, and VEGF with LODs from 5 to 50 fg mL^−1^ [96] and prediction of mucositis reaction from oral cancer treatment through detection of cytokines; tumor necrosis factor α (TNF-α), IL-6, Interleukin-1β (IL-1β), and c-reactive protein (CPR) at 10–50 fg mL^−1^ LODs [101] are good examples of clinical applications of MB-based systems. Furthermore, Krause et al. developed a sandwich immunoassay on inkjet-printed electrodes with greatly reduced assay times to achieve clinically-relevant 5 pg mL^−1^ LODs for IL-6 and IL-8, trading off sensitivity for speed, to achieve a clinically promising system for quick diagnosis [98]. Otieno et al. then modified this microfluidic device with an online protein capture chamber, where the analytes are separated from the serum in an isolated compartment with Mb-Ab_2_, before being introduced into the detection channel with the electrodes [99]. Otieno et al. further detected parathyroid hormone-related peptide (PTHrP) fragments which act as biomarkers for bone metastasis of breast and prostate cancer obtaining LODs of 3 fg mL^−1^,1000-fold less than the traditionally used immunoradiometric assay (IRMA) [100].

Extending the application of MB-enzyme conjugates further, Uliana et al. developed an elegant and inexpensive method to produce a fully disposable microfluidic electrochemical device (μFED) using SPE arrays for detection of breast cancer biomarker estrogen receptor alpha (ERα). Silhouette software was used for the designing of the electrodes and the patterned electrodes were transferred on to the vinyl sheets, followed by a vinyl mask and carbon ink from Henkel, which was poured on to the surface and then allowed to cure. Ag/AgCl ink was then deposited on the electrodes to obtain a μFED by sandwiching the SPEs using a double adhesive polystyrene card. Using this method, authors note that dozens of inexpensive ($0.20) devices could be constructed in just a few hours. Working electrodes were modified with DNA sequences known as estrogen response elements that are specific for Erα. A bioconjugate was developed using magnetic particles heavily labeled with ERα antibodies and enzyme labels of HRP were used to capture ERα in solution. The bioconjugate was injected into the μFED. Limits of detection of 10.0 fg mL^−1^ were achieved through amperometric detection with reduced incubation times [104].

While MBs are attractive labels to reach ultrasensitive recognition of proteins, the synthesis of MBs is a cumbersome process with issues in reproducibility, bioconjugation, and characterization. Moreover, when integrated into microfluidic devices, the bulky MBs are often found to block the tubing, requiring additional cleaning or periodic replacement of the tubes [92].

**Figure 7:**
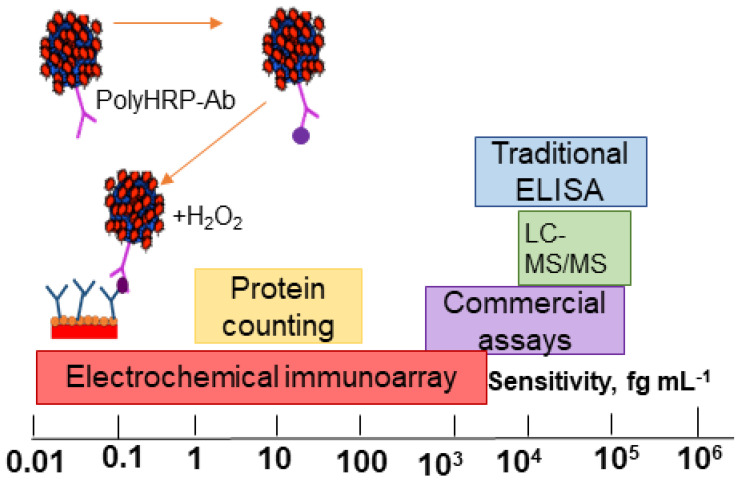
Comparison of the sensitivity of the new electrochemical immunoassay with poly-HRP and AuNP-modified carbon electrodes developed by Dhanapala et al. with other protein detection methods currently available. Reprinted with permission from [17], copyright 2020 American Chemical Society.

**Figure 8:**
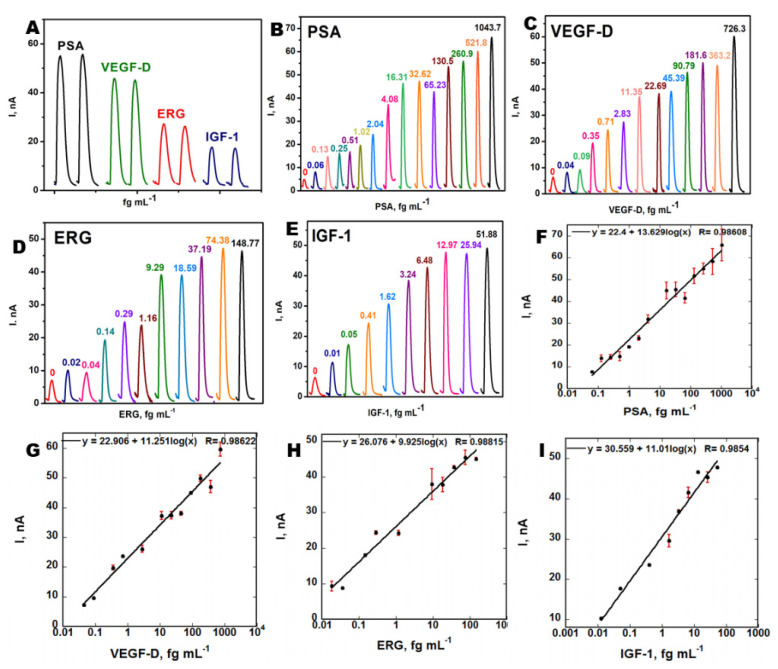
Calibration plots obtained with (**A**) duplicate amperometric peaks of PSA, VEGF-D, ERG, and IGF-1 showing reproducibility; (**B–E**) show amperometric peaks obtained for each biomarker standard; and (**F–I**) show the calibration plots with dynamic ranges from 0.013 to 1000 fg mL^−1^. Reprinted with permission from [17], copyright 2020 American Chemical Society.

Recent developments in producing multi-enzyme tags for detection of printed electrodes include the use of the polymerized form of enzymes, particularly HRP (poly-HRP) [105,106]. These polymers are conjugated with streptavidin molecules to bind with biotin-Ab_2_ via streptavidin–biotin interactions. Commercially, streptavidin-poly-HRP conjugates, such as strep-poly-HRP20, strep-poly-HRP40, and strep-poly-HRP80, are readily available. These conjugates contain five homopolymers, each carrying either 20, 40, or 80 HRP molecules covalently coupled with multiple streptavidin molecules [107]. Our group used strep-poly-HRP on a disposable, patterned array made from gold compact discs (CDs) for the detection of IL-6 with LOD 10 fg mL^−1^, at a cost of $0.2 per array [108]. Pingarrón et al. developed a magnetoimmunosensor on SPCE for detection of IL-6 coupled with carboxyl-functionalized magnetic microparticles for Ab_1_, but only reached a LOD of 0.4 pg mL^−1^ using poly-HRP80 [109]. The same strategy was later used for transforming growth factor-β (TGF-β) detection with LOD of 10 pg mL^−1^ [110]. They further modified the array with 4-carboxyphenyl-functionalized double-walled carbon nanotubes to improve the detection for multiplex proteins, TNF-α and IL-1β, with LODs 0.85 and 0.38 pg mL^−1^, respectively [111]. Meanwhile, our group recently published a multiplexed detection assay of four prostate cancer biomarkers, PSA, ETS-related gene protein (ERG), insulin-like growth factor-1 (IGF-1), and VEGF-D, using a system with strep-poly-HRP80 coupled with SPCE from Kanichi Ltd. modified with the glutathione-AuNP layers from Mani et al. [93]. Signal enhancement from high HRP/Ab_2_ ratio and the AuNP surface modification generated unprecedented sub-zeptomole LODs [17], representing the most sensitive multiplexed protein assay thus far reported (Figure 7). PSA, ERG, IGF-1, and VEGF-D LODs were 0.13, 0.063, 0.013, and 0.088 fg mL^−1^, respectively, accounting for less than 100 protein molecules (Figure 8).

### 2.3. Label-Free Detection

In the past decade, with the advancement of label-free methods on printed electrodes, advantages of label vs. label-free methods have been debated. While label-free methods offer ease of fabrication, less incubation time, and ease of automation, they still trail well behind labeled assays in sensitivity [112,113]. Label free methods use potentiometry, amperometry, voltammetry, conductometry, field-effect transistor (FET), and electrochemical impedance spectroscopy (EIS) as modes of detection [113,114]. Among these, EIS is the most frequently used mode due to its innate capability to sensitively measure any changes in the charging capacity, conductivity, or resistivity of an electrochemical surface when modified with biomolecules [113,114]. Davis et al. developed strategies for detecting C-reactive protein (CRP) on gold electrodes using aptamers, reaching LODs down to 300 pM using EIS measurements [115]. Aptamers are oligonucleotide or protein recognition probes that are selected for binding a target molecule from a large random sequence pool. Davis’ group has done interesting work on EIS-based label-free immunoassays that have catalyzed progress in unlabeled immunoassays and can be adapted to printed electrodes [116,117,118,119,120,121].

**Figure 9:**
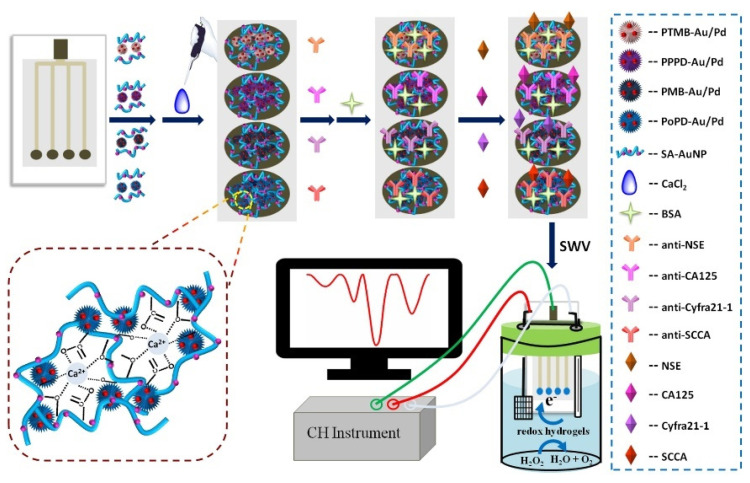
Label-free amperometric detection of four tumor biomarkers using specific hydrogel activation for each biomarker, with multiple working electrodes on a SPCE. Reprinted with permission from [122], copyright 2017 Elsevier B.V.

Zhao et al. developed a system with multiple working electrodes on a SPCE, each for specific target analytes coated with four different hydrogels activated by sodium alginate-Au nanoparticle (SA-AuNP) composites. They detected four proteins—CA-125, neuron-specific enolase (NSE), fragment antigen 21-1 (Cyfra21-1), and squamous cell carcinoma antigen (SCCA) at 0.0054 U mL^−1^, 2.3 pg mL^−1^, 5.5 pg mL^−1^, and 4.8 pg mL^−1^, respectively, using amperometric detection [122] (Figure 9). Many other works, in label-free protein detection, have attained clinically-relevant detection limits, but they have only detected single biomarkers and improvements are required for multiplexing [123,124,125,126].

## 3. Screen Printing

**Figure 10:**
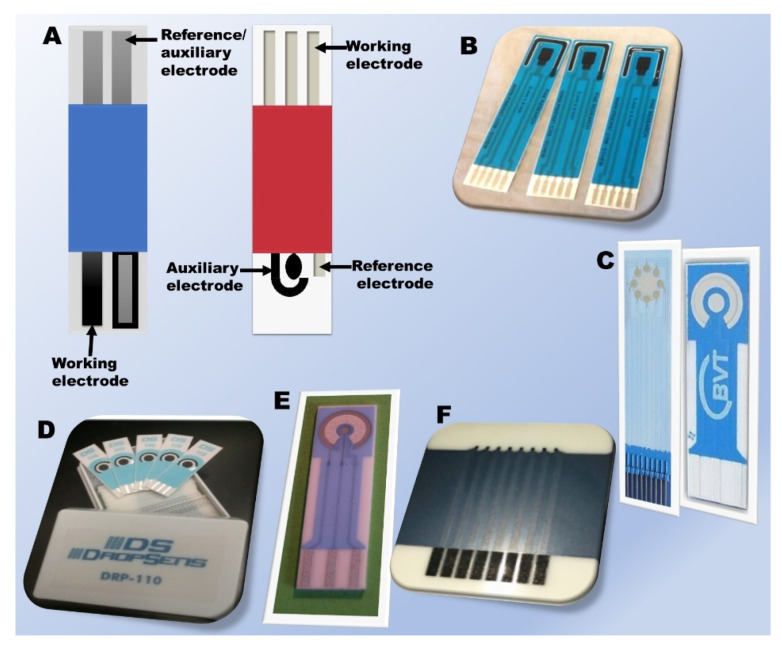
Screen-printed electrodes that are commercially available. (**A**) schematic diagram of generation one (left) and two (right) SPEs. (**B**) SPE from Pine Research including a carbon working electrode, a carbon counter electrode, and an Ag/AgCl reference electrode [133]. (**C**) left—AC9C Sensor array with graphite working electrode and Ag/AgCl reference electrode, right—AC1 Sensor with graphite working and auxiliary electrodes and Ag/AgCl Reference Electrode from BASi [135]. (**D**) metrohm SPE based on carbon, gold, platinum, silver, or carbon nanotube inks [132]. (**E**) PalmSens electrodes with same material working and counter electrodes (C, Pot, Ag, Au or Au-Pt alloy) and reference electrode made from Ag or Ag/AgCl [134] and (**F**) SPCE from Kanichi Research Services Ltd. housing eight working electrodes [136].

Screen printing employs a viscous ink or paste being passed over a screened mesh using a blade (also known as a squeegee) onto a substrate service [127]. This technique is also used for printing designs and labels on T-shirts. Several commercially-available inks are available from suppliers such as Dupont [128], Sun Chemicals [129], Acheson [130], and Tekra/Henkel [131]. Typically, for SPEs, the ink contains powdered silver gold, platinum, copper, or carbon along with adhesives and additives including resin, cellulose acetate, cyclohexanone, or ethylene glycol [30]. However, the formulation of the conductive pastes is typically not released due to its commercial value [127]. Substrates for thin-film electronics include plastics (polyester films, polyvinylchloride, polycarbonate, etc.), alumina, glass, ceramic, and paper. Several recent reviews outline the advantages and disadvantages of these inks and substrates [30,127]. Ready-to-use SPEs can also be purchased from commercial suppliers including Metrohm [132], Pine Research, [133] PalmSens [134], BASi [135], and Kanichi Research Services Ltd. [136] (Figure 10B–F).

First-generation SPEs included working and reference electrodes. Second-generation included the three-electrode configuration of working, reference, and auxiliary electrodes [30,34] (Figure 10A). Using these configurations, several groups have developed single analyte detection platforms. Without prior surface treatment of the SPE, Tallapragada et al. recently developed an immunosensor for the detection of breast cancer biomarker human epidermal growth factor receptor-2 (HER-2) [137]. Full sandwich immunoassays were developed on their previously designed SPE [138], which consisted of carbon paste working and auxiliary electrodes, and an Ag/AgCl paste reference electrode. Incubation times were consistent with ELISA and there was no improvement to total assay time. The redox reaction between the streptavidin-conjugated HRP label and 3,3′5,5′-tetramethylbenzidine was measured using cyclic voltammetry at a scan rate of 50 mV s^−1^ to quantify HER-2 in solution. The authors noted that it was the biotin–avidin chemistry that facilitated detection of HER-2 into the low nanogram levels of the detection limit of 4 ng mL^−1^ [137].

### 3.1. Nanoparticle Surface Coatings of SPEs

Enhanced performance in terms of sensitivity and LOD has been achieved on coated SPEs with nanoparticles [25]. These nanoparticles increase the surface roughness greatly, enhance the electrochemical surface areas, and allow for attachment of large quantities of capture antibodies for target analytes. Shana Kelly’s group has done a lot of interesting research on nanostructured modifications of electrode surfaces to show how nanostructured surfaces could be tuned to achieve high sensitivity. They detected carbohydrate antigen 125 (CA-125) down to 0.1 U mL^−1^ by optimizing the size of the chip sensors without using the conventional sandwich platform or labels, and this is a classic example of how much the surface modifications could enhance sensitivity even without further amplification strategies. Their findings indicate larger surface areas hinder reaching ultrasensitive measurements and suppression of the background signal can easily be achieved by minimal surface area [139]. We have already presented many examples of signal amplification by surface modification of SPE’s by nanoparticles [17,79,90,91,93,94,95,96,97,98,99,100,101,102,103,109,110,111]. A few more examples are included in this section.

Electrochemical deposition by Chan et al. generated a graphene-gold nanocomposite on the surface of Dropsens SPE for the detection of cancer biomarker, CEA [140]. A sandwich immunoassay was constructed on this nanocomposite surface using HRP as the redox label and H_2_O_2_ as an activator, and cyclic voltammetry was used to determine the concentration of CEA in the sample. The LOD was 0.28 ng mL^−1^ [140]. Incubation steps here were also consistent with conventional ELISA. Suresh et al. modified the surface of a SPE using chitosan and gold nanoparticles (AuNPs) for the detection of PSA [141]. A sandwich immunoassay protocol was followed with HRP as the enzyme, but they amplified the signal using methylene blue as a redox mediator. Electrochemical detection of PSA was monitored by cyclic voltammetry and square wave voltammetry. A dynamic range of 1–18 ng mL^−1^ was observed with LOD 1 pg mL^−1^ [141].

Giannetto et al. demonstrated a competitive immunosensor for determination of bladder cancer biomarker p53 protein on a DropSens SPE modified with a carbon nanotubes/AuNP composite [142]. Protein p53 was immobilized on the modified SPE followed by a single anti-p53 mouse monoclonal antibody that recognizes both wild-type and mutant p53. After immunocompetition in the sample, an alkaline phosphatase-conjugated reading antibody was added for electrochemical detection. In urine, this nano-modified SPE exhibited a wide linear range, from 20 pM to 10 nM, with LOD of 14 pM for p53 protein [142]. DropSens SPE was also used by Marques et al. for the simultaneous detection of breast cancer biomarkers CA 15-3 and HER-2 [143]. The SPE was coated with AuNP. Then the authors enzymatically deposited metallic silver using alkaline phosphatase [144]. Voltammograms were recorded to observe the electrochemical oxidation current of the enzymatically-deposited silver for LODs of 5.0 U mL^−1^ for CA 15-3 and 2.9 ng mL^−1^ for HER-2 [143].

### 3.2. SPEs and Molecular Imprinting

**Figure 11:**
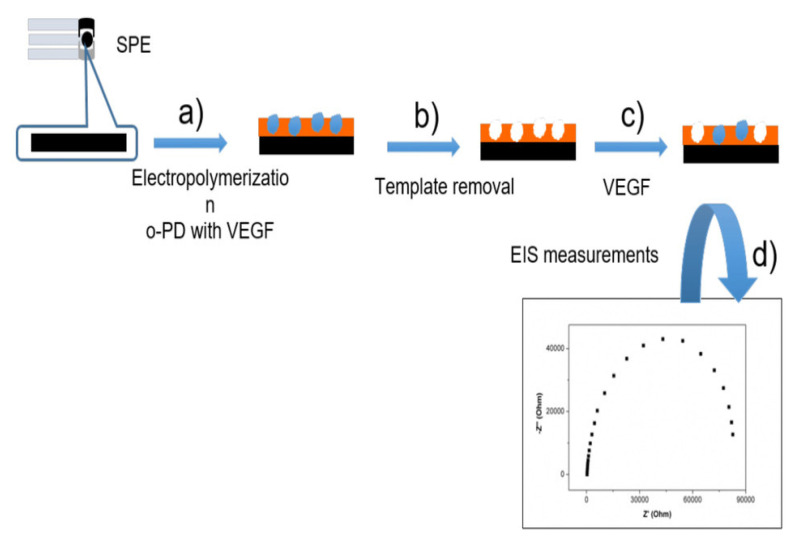
Schematic representation for the process of molecular imprinting (Bozal-Palabiyik et al.) through the electropolymerization of o-phenylenediamine (o-PD) for label-free detection. Reprinted with permission from [146], copyright 2019 Wiley-VCH Verlag GmbH and Co. KGaA, Weinheim.

SPEs have also served as the base for a molecular imprinted sensor [145]. Molecular imprinting involves developing affinity polymers for target analytes. The template or target analyte interacts during polymerization on the surface to form a polymerized binding site on the surface of SPE. Once the template is removed, it leaves the binding sites free for the subsequent capture of the imprinted analyte [145]. Bozal-Palabiyik et al. developed a molecularly-imprinted polymer on the surface of a SPE for cancer biomarker VEGF [146]. They used label-free impedance to detect VEGF with a dynamic range from 20 to 200 pg mL^−1^ and a limit of detection of 0.08 pg mL^−1^ [146] (Figure 11). Gomes et al. developed an electropolymerized SPE with amine-substituted benzene rings as monomers and specifically charged monomers for creating protein imprinting materials for breast cancer biomarker CA 15-3 [147]. The commercial Au SPE from DropSens consists of gold working and auxiliary electrodes, and reference and electrical contacts made of silver. Using this sensor, Gomes et al. demonstrated a linear dynamic range of 0.25 to 20.00 U mL^−1^, with detection limit of 0.05 U mL^−1^, for CA 15-3, from square wave voltammograms in diluted serum, with only 15 min incubation between sensor and solution of CA-15-3 [147].

### 3.3. SPE in μPADs

**Figure 12:**
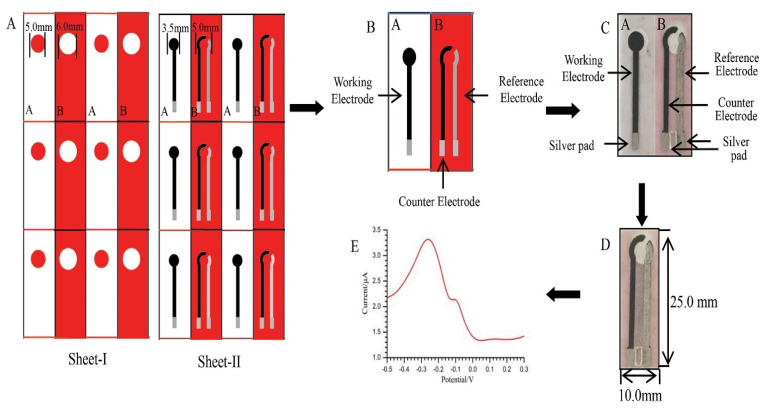
The fabrication of the paper-based electrodes by Fan et al. (**A**) Sheet I and II indicate the screen-printed wax patterns. Paper A and B of Sheet I contain the sample zone (5.00 mm) and the auxiliary zone (6.00 mm), which were designed for the carbon counter, working, and Ag/AgCl reference electrodes. Sheet II contains Paper A and B with patterns for the above three electrodes. (**B**) Represent a full screen-printed electrode system with all the three electrodes: counter, working, and reference. (**C**) A photograph of the produced electrode system with a silver pad. (**D**) Folded electrode. (**E**) A typical signal from the DPV used for the detection of the immunosensor. Reprinted with permission from [152], copyright 2019 Elsevier B.V.

Microfluidic electrochemical immunosensing was pioneered by Heineman in the early 2000s [148,149], and paper microfluidic devices (μPADs) were first reported by Whiteside’s group to improve the ease of operation and ability to work without external pumps [150]. SPEs on the surface of the paper were explored by several groups [151]. Fan et al. fabricated a SPE on the surface of paper modified with graphene oxide/thionine/gold nanoparticles (rGo/Thi/AuNPs) for detection of CA 125 [152]. Using custom-designed screens, electrodes were patterned onto wax-printed paper. Carbon ink was used for both working and counter electrodes, and Ag/AgCl ink was used for a reference. The patterned paper was folded with double-sided tape for use. The (rGo/Thi/AuNPs) were used for not only antibody immobilization but also signal amplification. The detection principle followed that the CA 125 antigen could decrease the current response of thionine, which corresponded to the concentration of CA 125 [152]. Using differential pulse voltammetry, the immunosensor displayed a linear range from 0.1 to 200 U mL^−1^ with LOD of 0.01 U mL^−1^ [152] (Figure 12).

## 4. Inkjet Printing

**Figure 13:**
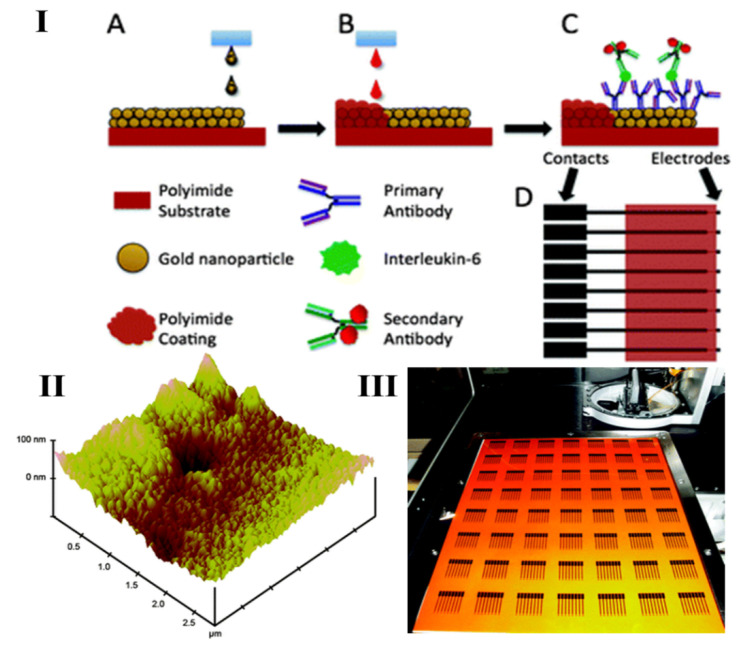
The inkjet-printed electrode immunoassay. (**I**) Schematic diagram of the immunoassay procedure indicating (**A**) AuNP inkjet printing on to the Kapton polyimide substrate, (**B**) insulation with poly(amic) acid, (**C**) immunoassay build-up, and (**D**) the electrode surface with AuNP patterns in black and insulating poly(amic acid) patterns in orange. (**II**) Tapping mode Atomic Force Microscopy (AFM) image of the AuNP working electrode surface. (**III**) A total of 56 eight-electrode arrays printed on a Kapton surface by Dimatix Materials Printer. Reprinted with permission from [31], copyright 2012 The Royal Society of Chemistry.

In efforts to decrease the cost of electrochemical arrays, our team has developed inkjet-printed arrays [31,98]. Inkjet printing offers several advantages over screen printing in that it is a contactless method for fabrication that does not require a stencil or template [32,35,153]. Design patterns can be developed using digital software patterns that can be sent to the relatively simple materials inkjet printer in a similar manner to using an office inkjet printer. Several groups developed simple techniques using a regular office printer with slight modifications [154,155]. However, for further control and precision, more sophisticated material printers are available at a reasonable cost [31,98]. Commercially available inks exist from the same suppliers. Several groups have synthesized their own custom inks. Recent reviews on inkjet-printed platforms provide additional details on the printing process and inks available [31,98] (Figure 13).

Using a custom-fabricated ink made from dodecane thiol-protected gold nanoparticle in toluene, we printed arrays of gold electrodes on the surface of a heat-resistant polyimide Kapton plastic sheet as a platform for cancer detection [31,98]. The inkjet-printed array was printed using a FUJIFILM Dimatix Materials Printer. With less than 2 mL of ink, dozens of arrays can be printed. Electrode arrays were insulated with a printed overcoating of poly(amic) acid, a precursor that converts to Kapton when heated. The inkjet-printed working electrodes had reproducible surface areas with relative standard deviation (RSD) < 3% [56,57]. The material cost was $0.2 [31,98]. Using this process, the methods could easily be scaled up using industrial-sized inkjet printers [31,98]. For immunoassay applications, the electrode arrays were heated at 200 °C for 30 min, cleaned in sulfuric acid, and sensors coated with a self-assembled monolayer (SAM) of mercaptopropionic acid (MPA) to introduce carboxyl groups for cross-linking capture antibodies to the working electrodes. Arrays were used in microfluidic platforms with external counter and reference electrodes. Using this strategy, we reported the first ultrasensitive multiplex peptide assay to measure intact parathyroid hormone-related peptide PTHrP 1-173, as well as circulating N-terminal and C-terminal peptide fragments [100].

Fully inkjet-printed electrochemical sensors with integrated counter and reference electrodes have also been developed [156,157]. We recently reported a fully disposable inkjet-printed electrochemical platform for HER-2 [158]. The electrochemical sensor platform consisted of an inkjet-printed gold working eight-electrode array (WEA) and counter electrode, along with an inkjet-printed silver electrode that was chlorinated with bleach to produce an Ag/AgCl reference electrode. The array sensors were treated with MPA to allow for conjugation of capture antibodies. Once decorated with capture antibodies, the arrays were used in a microfluidic channel to implement a sandwich immunoassay on the surface of the WEA, following a simultaneous injection of target protein, biotinylated antibody, and polyHRP label [158]. Clinically-relevant LODs of 12 pg mL^−1^ were achieved with an assay time of only 15 min [158].

## 5. Challenges for Printed Electrodes in Microfluidic Assays

Microfluidics coupled with printed electrodes have been in the forefront of emerging electrochemical microsystems, synergistically enhancing inherent features of each other in lab-on-chip platforms for POC. Despite its success in production of wearables and self-powered devices [25,159,160], these approaches need to fill gaps in technical and clinical aspects to be used in commercialized cancer detection platforms.

Adaptation of microfluidics with printed electrodes into clinical platforms is relatively new as it requires a multi-disciplinary foundation of chemistry, physics, engineering, medicine, and biology [161]. Fabrication of microfluidic devices involve a multistep process including designing, material selection, processing and surface treatment with each step having their own technical issues [162]. Fabrication protocols such as photolithography, polymer molding, lase ablation, lamination, soft lithography, and, most recently, 3D printing [163] are available for chip production, but bottlenecks at the closing stages of the microstructure which may introduce defects in design, and is also a time-consuming process [163,164]. Integration of printed electrodes into microfluids also has its own set of technical difficulties, such as complex initial designs where all aspects of the final platform must be optimized such as mixing, binding, separation, washing, and detection. Incorporation of electrodes on microfluidic chips must avoid channel deformations and electrode damage. New materials and fabrication protocols are being introduced to solve these technical issues. Replacement of the silicon- and glass-based chips with polydimethylsiloxane (PDMS) provided several advantages, including mechanical flexibility, biocompatibility, electric insulation, optical transparency, ease of handling and manipulation, and low cost [28,164,165,166]. PDMS chips have challenges such as leaching, channel deformation, evaporation, sample adsorption, low acid-base resistivity, and hydrophobic recovery, providing a challenge to use in routine clinical practice [165]. Novel fabrication protocols, such as 3D printing and nanofabrication, are now emerging as solutions to overcome some of the traditional complications involved in microfluidics and to extend it to a more commercialized platform [159,163,165,167].

Reliable future diagnostics will require rapid detection of a panel of biomarkers at low cost, in clinically-relevant ranges. We discussed above many examples where such systems have been fabricated, especially for multiplexed detection of two to four biomarkers. Multiplexing beyond four biomarkers for biomedical samples remain a challenge, but not impossible. These issues will need to be addressed through creative engineering to produce ideal POC platforms for protein panels.

## 6. Conclusions

We have summarized above many different strategies of fabricating printed electrodes for the detection of cancer biomarker proteins in clinically-relevant ranges. Our focus was on inkjet-printed and SPEs, with the majority of examples covering the latter due to its widespread use in immunosensor development. Advantages include low cost, speed, and ease of fabrication in sensor designs tailored specifically for the microfluidic system being developed. Disadvantages include lack of reproducibility of sensor areas in arrays in some cases, but this can often be overcome with surface area measurements. Electrochemical ELISA-based approaches used in most cases can lead to low cost, ultrasensitive multiplexing of immunoassays, with excellent potential for future clinical applications. One particular electrochemical immunoassay holds the current record for the world’s lowest protein LODs in serum, in the sub-fg mL^−1^ range [17]. Electrochemical detection has some limitations for POC applications; however, since individually addressed electrode sensors and a multiplexed electrochemical work station are needed, as opposed to detection by ECL and chemiluminescence, which do not require sophisticated electronic measurements and can be measured with a CCD camera [164]. Automation is also easier with the ECL and CL detection.

Most of the immunosensors discussed have used a variety of labels to achieve ultrasensitive multiplexed detection, with some new research trending towards label-free detection methods. Though unlabeled strategies have improved in sensitivities over the years, they still trail behind labeled biomarkers that have reached record LODs down to 0.02 fg mL^−1^ and below, enabling detection of fewer than 100 protein molecules per sample [17]. Multi-enzyme labeling coupled with nanostructured electrode surfaces are key factors for electrochemical signal enhancement and higher sensitivity. One challenge in SPE immunoassays is a fully automated system, preferably with every component integrated into a single chip. Up to now, only semi-automated units have been exploited with washing, sample preparation, and reagent addition being stumbling blocks in the path to automation.

Despite the delay in automation, immunoassays based on printed electrodes have a huge potential in cancer diagnostics, especially with the ability to be adopted into multiplex detection platforms, which is essential for accurate cancer detection and staging. Unfortunately, current research advances in immunoarrays have not reached active clinical practice for the detection and monitoring of cancer. The transition to clinical use will most likely require commercial development. We hope that the printed electrode immunoassays will overcome this barrier in the near future and be used in future widespread clinical cancer diagnostics.

## Figures and Tables

**Table 1:** FDA approved protein biomarkers [3,5,14,15].

Biomarker		Sample	Cancer Type	Clinical Use	Assay
Abbreviation	Name				
Free PSA/fPSa	Free Prostate Specific Antigen	Serum	Prostate	Benign Hyperplasia vs. Cancer diagnosis	Immunoassay
tPSA	Total PSA	Serum	Prostate	S, M	Immunoassay
cPSA	Complex PSA	Serum	Prostate	S, M	Immunoassay
p63	Transformation-related protein 63	FFPE tissue^†^	Prostate	S, M	Immunohistochemistry
TG	Thyroglobulin	Serum	Thyroid	S, M	Immunoassay
EGFR	Epidermal growth factor receptor	Colon tissue	Colon	Pre	Immunoassay
CEA	Carcinoembryonic antigen	Serum	Colon	M	Immunoassay
MW CEA	High molecular weight CEA	Urine	Bladder	M	Immunofluorescence
FDP (AMDL-ELISA DR-70)	Fibrin/fibrinogen degradation products	Urine/Serum	Bladder	M	Immunoassay
NMP/22	Nuclear matrix protein 22	Urine	Bladder	S, M	Immunoassay
BTA	Bladder tumor antigen	Urine	Bladder	M	Immunoassay
HER2	Human epidermal growth factor receptor	Serum	Breast	M	Immunohistochemistry
CA15-3 *	Carbohydrate antigen 15-3	Serum, plasma	Breast	M	Immunoassay
CA27-29 *	Carbohydrate antigen 27-29	Serum	Breast	M	Immunoassay
HER/NEU	Human epidermal growth factor receptor 2	FFPE tissue^†^	Breast	P, Pre	Immunohistochemistry
ER	Estrogen factor	FFPE tissue^†^	Breast	P, Pre	Immunohistochemistry
PR	Progesterone factor	FFPE tissue^†^	Breast	P, Pre	Immunohistochemistry
AFP*	α-fetoprotein	Serum, plasma, amniotic fluid	Testicular	St	Immunoassay
β-hGC *	Human chorionic gonadotropin-β	Serum	Testicular	St	Immunoassay
AFP-L3%	α-fetoprotein L3% isoform	Serum	Hepatocellular	P	HPLC, microfluidic capillary electrophoresis
KIT	Receptor Tyrosine Kinase	FFPE tissue^†^	Gastrointestinal stromal tumors	Pre	Immunohistochemistry
CA 19-9 *	Carbohydrate antigen 19-9	Serum	Pancreatic	M	Immunoassay
CA 125 *	Carbohydrate antigen 125	Serum	Ovarian	M	Immunoassay
HE4	Human epididymis protein 4	Serum	Ovarian	M	Immunoassay
OVA1 (Multiprotein test	CA125, Apolipoprotein A1, Beta-2 microglobulin, Transferrin, Pre-albumin	Ovarian	Serum	P	Immunoassay

M—monitoring, S—screening, P—prognosis, Pre—prediction of therapy, St—staging, *—Glycoproteins, FFPE tissue^†^—formalin-fixed paraffin-embedded (FFPE) tissue slides [23].
